# Biomarkers for Monitoring Pre-Analytical Quality Variation of mRNA in Blood Samples

**DOI:** 10.1371/journal.pone.0111644

**Published:** 2014-11-04

**Authors:** Hui Zhang, Vlasta Korenková, Robert Sjöback, David Švec, Jens Björkman, Mogens Kruhøffer, Paolo Verderio, Sara Pizzamiglio, Chiara Maura Ciniselli, Ralf Wyrich, Uwe Oelmueller, Mikael Kubista, Torbjørn Lindahl, Anders Lönneborg, Edith Rian

**Affiliations:** 1 DiaGenic ASA, Oslo, Norway; 2 Laboratory of Gene Expression, Institute of Biotechnology, Academy of Sciences of the Czech Republic, Prague, Czech Republic; 3 TATAA Biocenter, Gothenburg, Sweden; 4 AROS Applied Biotechnology AS, Aarhus, Denmark; 5 Fondazione IRCCS Istituto Nazionale dei Tumori di Milano, Milano, Italy; 6 Qiagen GmbH, Hilden, Germany; The University of Hong Kong, Hong Kong

## Abstract

There is an increasing need for proper quality control tools in the pre-analytical phase of the molecular diagnostic workflow. The aim of the present study was to identify biomarkers for monitoring pre-analytical mRNA quality variations in two different types of blood collection tubes, K_2_EDTA (EDTA) tubes and PAXgene Blood RNA Tubes (PAXgene tubes). These tubes are extensively used both in the diagnostic setting as well as for research biobank samples. Blood specimens collected in the two different blood collection tubes were stored for varying times at different temperatures, and microarray analysis was performed on resultant extracted RNA. A large set of potential mRNA quality biomarkers for monitoring post-phlebotomy gene expression changes and mRNA degradation in blood was identified. qPCR assays for the potential biomarkers and a set of relevant reference genes were generated and used to pre-validate a sub-set of the selected biomarkers. The assay precision of the potential qPCR based biomarkers was determined, and a final validation of the selected quality biomarkers using the developed qPCR assays and blood samples from 60 healthy additional subjects was performed. In total, four mRNA quality biomarkers (USP32, LMNA, FOSB, TNRFSF10C) were successfully validated. We suggest here the use of these blood mRNA quality biomarkers for validating an experimental pre-analytical workflow. These biomarkers were further evaluated in the 2^nd^ ring trial of the SPIDIA-RNA Program which demonstrated that these biomarkers can be used as quality control tools for mRNA analyses from blood samples.

## Introduction

Many promising RNA biomarkers have not proved to be clinically useful [1], [2] due either to analytical or pre-analytical errors (i.e. poor specimen quality caused by incorrect handling during storage or transport) or both. A number of researchers have found that in blood, alteration of gene expression starts almost immediately at the time of phlebotomy due to *ex* *vivo* gene induction, down-regulation, or RNA degradation [3], [4]. These unwanted pre-analytical effects have a direct effect on analytical results, particularly when sensitive methods like quantitative (q) PCR, the principal method for analysis of RNA species, are used. A considerable effort has been made to improve reliability of the analytical phase of qPCR, and a comprehensive set of guidelines have been generated (the MIQE guidelines) [1], by now widely accepted by the research community [5]–[7]. Improving the analytical precision of qPCR has further revealed the importance of controlling pre-analytical variables which may affect analytical results. In the past few years, the effort to control pre-analytical errors has increased [8]–[12]. The work presented here is the result of a large collaboration within the European FP7 project SPIDIA: Standardization and Improvement of generic Pre-analytical Tools and Procedures for In-vitro Diagnostics [13]. One of the main goals of SPIDIA has been to develop biomarkers which enable monitoring of changes within a biospecimen after collection, and during transport, and storage. These biomarkers are intended to serve as quality control tools in research and in clinical laboratories. Validated quality biomarkers should be a critical tool in for evaluation of the processing of any biospecimen, and, when used routinely, allow for proper inclusion or exclusion of a specimen or results from that specimen. Improper treatment of specimens may yield degraded RNA or cause activation or down-regulation of gene expression which directly influences the quantification of a specific RNA species. These effects can lead to an erroneous estimation of target mRNA copy number and vastly increase variability of the overall results. The effect of RNA quality and quantity on reverse transcription qPCR (RT qPCR) results can be very pronounced and therefore significantly influence interpretation of gene expression in these specimens [14]–[16].

At present, there are only a few appropriate quality control tools available. The standard approach is to assess RNA integrity [15] based on the measurement of 28S and 18S ribosomal RNA ratios. These methods (RIN score, Agilent BioAnalyzer; RQI, Experion, Bio-Rad) reflect the integrity of the dominant ribosomal RNA but not necessarily the integrity or amount of the relevant mRNA species [17], [18]. Other molecular methods, therefore, have been developed to assess mRNA quality.

One such molecular method is the 3′/5′ assay [19]. In this method, two qPCR amplicons are designed to target either end of a given transcript. Since amplification of the amplicon at the 5′-end will only work if the transcript is intact, the comparison of the cycle of quantification (Cq) of 3′ and 5′ assays will reveal the integrity of the transcript. Another method is the short/medium/long assay [20]–[22] which compares the Cq values of amplicons with different lengths using one common forward primer. The longer amplicon will have a higher Cq if the mRNA is degraded.

In this article, we utilize previously described methods and present the process and results leading to the identification and validation of a set of candidate biomarkers for monitoring pre-analytical variation of mRNA in human blood samples collected in EDTA and PAXgene tubes. Validation was carried out with blood specimens collected from a new, independent cohort of 60 apparently healthy subjects. In total, four RNA quality biomarkers were successfully validated.

## Materials and Methods

### Study design

Blood specimens were collected in either EDTA or PAXgene tubes and the RNA was isolated immediately after collection and at other subsequent time points. mRNA quality biomarker candidates were identified using microarray analysis by selecting RNA species demonstrating the greatest changes in quantity over the storage period studied. The selected biomarkers were further verified with qPCR in a pre-validation study, and the precision of the qPCR assays developed for each biomarker was determined. Finally, the biomarkers were validated with qPCR in a large cohort of samples derived from healthy subjects.

### Blood collection and ethics statement

Whole blood was collected from each healthy, adult subject into K_2_EDTA (EDTA) tubes (BD, cat. no. 367525) and PAXgene Blood RNA Tubes (PAXgene) (PreAnalytiX) using standard phlebotomy technique. The number of tubes and volume of blood collected for each study are described in Table 1. After approval by Norwegian south east regional committee for medical and health research ethics (REC South East), all participants signed a written informed consent before participating in the study in accordance with the Helsinki declaration.

**Table 1:** Summary of sample material and extraction methods for all studies.

Study	Tubetype	Temperature	Incubationtime (hr)	Number ofsubjects	Number oftubes persubject	Bloodvolumeper tube	Extractionmethod
**Microarray**	EDTA	4°C, RT	0, 24, 48, 72	3	8	2 mL	EDTA protocol 11)
	PAXgene	4°C, RT	0, 24, 48, 72	3	8	2.5 mL	PAXgene protocol2)
**Precision**	EDTA	RT	0, 24	8	4	2 mL	EDTA protocol 1
	PAXgene	35°C	0, 48	4	8	2.5 mL	PAXgene protocol
**Prevalidation**	EDTA	RT	0, 2, 6, 24, 48, 72	6	6	2 mL	EDTA protocol 1
	PAXgene	RT	0, 24, 48, 72	8	4	2.5 mL	PAXgene protocol
		35°C	0, 24, 48, 72	5	4	2.5 mL	
**Extended** **validation**	EDTA	RT	0, 24, 48	60	3	2.5 mL	EDTA protocol 23)
	PAXgene	30°C	0, 48, 72	60	3	2.5 mL	PAXgene protocol

1) Acidic organic phenol extraction and silica membrane clean up.

2) PAXgene Blood RNA Kit handbook version 2 protocol (PreAnalytiX, Hombrechticon).

3) After the indicated storage time, the EDTA sample was transferred into PAXgene Blood RNA tube followed by PAXgene Blood RNA extraction procedure.

### RNA extraction

RNA from blood collected in PAXgene tubes was extracted using the PAXgene Blood RNA Kit (PreAnalytiX) according to the manufacturer’s instructions. Except for the validation study, RNA from EDTA blood was extracted with acidic organic phenol extraction and silica membrane clean-up. To reduce tube-related bias in the validation study, 2.5 mL of blood collected in EDTA tubes were transferred into PAXgene tubes at the indicated storage time and the RNA extracted with the PAXgene Blood RNA Kit. RNA was immediately stored in −80°C. RNA quantity was measured with Nanodrop spectrophotometer ND-1000 (Thermo Scientific) and each RNA integrity was measured with Agilent 2100 Bionalyser (Agilent Technologies). Information on the samples is included in Table 1.

### Microarray analysis

The gene expression analyses were performed according to the Affymetrix and NuGEN procedures. In brief, 50 ng of RNA labeled with the NuGEN labeling procedure, loaded onto the Affymetrix GeneChip U133 Plus 2.0 cartridge, and hybridized for 16 h. The arrays were washed and stained in the Affymetrix Fluidics Station and scanned using the Affymetrix GeneChip Scanner.

### qPCR assay design

Primer design was performed with Primer BLAST [23] and probe design with Beacon Designer (PREMIER Biosoft International). All assays were designed to span at least one intron and/or to have one primer covering an exon/exon boundary. For degradation markers, short/medium/long (S/M/L) amplicon length and 3′/5′ primer assays were used to select final candidate biomarkers of mRNA degradation. For up- and down-regulation biomarkers, only short amplicons were utilized. Criteria for the assays were: linearity with 5-log dynamic range and LC480 error<0.2 (LC480 error is the mean square error of the single data points fit to the regression line), efficiency (80%<efficiency<105%), specificity (no amplification of gDNA or ≥5 cycles difference between target and genomic Cq-value) and no amplification of No Template Controls (NTCs). All assays (10 µl reaction volume) were evaluated with the QuantiTect SYBR Green PCR Kit and QuantiTect Probe PCR Kit (Qiagen) on the LightCycler 480 (Roche), Realplex (Eppendorf), and Rotor-Gene 6000 (Corbett Research/Qiagen). The QuantiTect SYBR run protocol was as follows: activation @ 95°C for 15 min; amplification @ 95°C for 15 s, 57°C for 30 s; and 72°C for 30 s (45 cycles); melting curve temperature range 60–95°C. The QuantiTect Probe run protocol was as follows: activation @ 95°C for 15 min, amplification @ 95°C for 15 s and 61°C for 60–120 s (50 cycles). All assays were initially evaluated with SYBR green chemistry to test the primers. After approval of the primers, a hydrolysis probe was designed (shared probe for the assays in the S/M/L system) and evaluated as described for the primers. Three-prime/five-prime (3′/5′) and short, medium, and long assays were designed to span 80–160 bp, 200–370 bp, and 400–550 bp respectively. The annealing temperature was set to 60°C *in* *silico* and evaluated experimentally with a temperature gradient test. Optimal annealing temperature was determined to be 57°C for SYBR chemistry primers and 61°C for hydrolysis probe chemistry. Information on all primers and their validation is included in Table S1.

### cDNA synthesis

Reverse transcription in precision, pre-validation, and validation studies were performed using ABI High Capacity cDNA Reverse Transcription (Life Technologies) with random hexamers. In qPCR assay development, SuperScript III First-Strand Synthesis System (Life Technologies) was employed with random hexamers. Manufacturer’s instructions were followed using 1 µg RNA per sample in 10 µl and cDNA was diluted 1∶10 prior to PCR setup.

### qPCR analysis

For biomarker validation, all assays were run using QuantiTect Probe PCR Kit (Qiagen) with a protocol as follows: activation @ 95°C for 15 min; amplification @ 95°C for 15 s and 61°C for 90 s, (40 cycles), 2 µl of cDNA in 20 µl of the total volume in 7900HT Fast Real-Time PCR System (Applied Biosystems). Each sample was run in technical triplicates. Candidate biomarker assays determined from each type of collection tubes were used on blood collected in the respective tubes. “No reverse transcription” and NTC controls were included for all assays throughout the process. Reference gene candidates were checked on both EDTA and PAXgene samples using Normfinder (GenEx, MultiD Analyses). All data were pre-processed in GenEx Enterprise (MultiD Analyses).

### Statistical analysis

#### A) Microarray data

The microarray data set (see File S1) was analyzed using two different normalization methods: the Robust Multi-array Average (RMA) method [24] without background correction and the quantile normalization procedure implemented in RMA. The statistical analyses were performed using the R software version 2.10 [24] and Bioconductor software version 2.5 [25].

#### B) Validation study

Starting from preliminary results from a pre-validation study (see File S2), we planned to collect blood samples from 60 apparently healthy subjects which would allow detection of gene expression changes between time points with a power of 80% and a nominal two-sided significance level of 0.05 [26]. To validate the selected biomarkers, a mixed model [27] was implemented with data from 60 subjects. The dependent variable, –ΔCq, was modeled as a function of ‘time’ (fixed factor) and ‘donor’ (random factor). For the up- and down-regulated EDTA biomarkers, the Cq values of short assays were utilized for the calculation of the corresponding –ΔCq at each time point as follows: ΔCq marker = Cq marker–Cq meanref (Cq meanref = mean of the Cq values from the short assay of reference genes). For the PAXgene degradation biomarkers, the ΔCq at each time point was calculated as follows: ΔCq marker S/M = Cq short assay–Cq medium assay.

Additionally, for each EDTA biomarker, we evaluated the relevance of the expression changes between T_0_ and the other two time points by computing the 95% simultaneous confidence interval (SCI) of the Log_2_ of the relative quantity (RQ) [i.e. Log_2_(RQ) = −ΔΔCq; where ΔΔCq = ΔCq marker(T_x_)−ΔCq marker (T_0_)]. This approach [28] takes into consideration the simultaneous determination of all the markers on the same set of subjects. Following the conventional two-fold threshold rule, we considered the down-regulation and the up-regulation of a specific biomarker as statistically relevant if the upper limit of the 95% SCI of Log_2_(RQ) was ≤−1 and the lower limit of the 95% SCI of Log_2_(RQ) was ≥1. The analysis was carried out using SAS software v. 9.2 (SAS Institute Inc. Cary, NC).

## Results

### Microarray analysis

Biomarker candidates were identified by microarray analysis of RNA from blood collected in either EDTA or PAXgene tubes from three subjects. Tubes were stored at either room temperature (RT) or 4°C for up to 72 hours.

All probe sets with fold change >2 at the last time point were identified, resulting in 4982 unique probe sets representing 3748 unique genes. The probe sets common to all three subjects were identified, and from these, probe sets indicating higher or lower mRNA levels relative to T_0_ (both at RT and 4°C) were identified. For EDTA tubes kept at 4°C, a total of 36 genes with higher and 14 genes with lower mRNA levels were identified whereas the numbers were 1014 and 1407, respectively, for the RT EDTA samples. For PAXgene tubes, no genes showed higher relative mRNA levels at 4°C, whereas two were identified with lower relative signal levels. At RT, 12 and 1013 genes were identified with potentially higher and lower relative mRNA levels, respectively. Average signal from all three subjects for each probe set was calculated. Candidate genes with a linear and stable increase or decrease in signal intensity over time and a similar profile for all three subjects were selected. The 16 most significant candidate genes for blood collected in PAXgene tubes and the 8 most significantly lower and 16 higher expressed candidate genes for EDTA blood were selected (for a total 40 candidate genes). Microarray probe sets in the 3′ and 5′ end for each gene were identified.

### Biomarker assay development

For 15 of the identified 40 candidate gene biomarkers, qPCR assays were designed, tested against pre-set criteria (see Materials and Methods), and evaluated for specificities and sensitivities (see File S2, Figure S1). In addition, three reference genes, GAPDH, GUSB, and PPIB, were selected based on their stable expression levels in the microarray data. These reference genes were used for normalization of EDTA up- and down-regulated biomarkers. These candidate gene biomarkers and reference genes are listed in Table S1.

The qPCR assays were used to verify selected biomarker candidates. Candidate genes with a significant increase or decrease in the expression level with respect to T_0_ and consistent expression changes over time for all subjects were further selected (see File S2, Figures S2, S3, S4, S5). Next, by excluding biomarker candidates with poor precision (see File S2, Table S2, Figure S6), a total of seven biomarkers were selected from pre-validation study.

### Validation of RNA quality biomarkers

The seven RNA quality candidate biomarkers included in the validation using a new cohort of 60 subjects were LMNA, TNF, FOSB, ATP2B4, TNRFSF10C, FAM126B, and USP32.

For each subject, blood collected in the PAXgene T_0_ tubes was incubated for 2 h at RT according to manufacturer’s instructions and stored *in* *situ* at −80°C or −20°C. For specimens collected in EDTA tubes, 2.5 mL of blood from the EDTA T_0_ tube was transferred to PAXgene tubes as soon as possible after blood collection stored as described above. Two additional EDTA tubes were incubated at RT, one for 24 h (T_24_) and one for 48 h (T_48_). For blood collected in PAXgene tubes, in addition to the T_0_ specimen, two additional PAXgene tubes were incubated at 30°C for 48 h (T_48_) and for 72 hours (T_72_) respectively. Since PAXgene tubes are validated for transport and storage of blood specimens for up to three days at 18°C–25°C, we considered the elevated storage temperature a relevant simulation of off-label, improper specimen handling.

**Figure 1:**
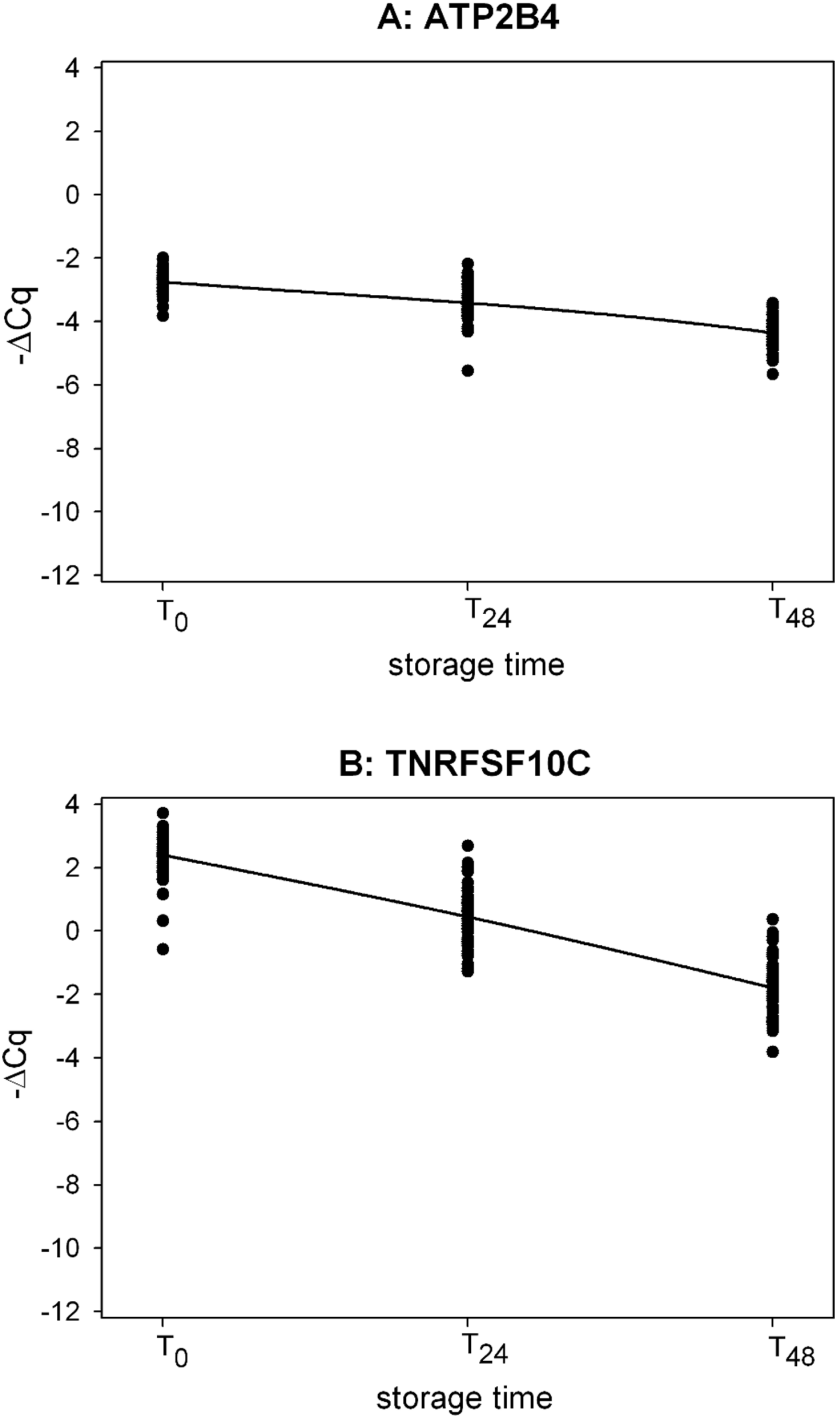
Time-course profile of EDTA down-regulation biomarkers in the validation study. 1A: ATP2B4_S (mixed model contrasts: T_24_ vs T_0_, p-value <0.0001; T_48_ vs T_0_, p-value <0.0001); 1B: TNFRSF10C_S (mixed model contrasts: T_24_ vs T_0_, p-value <0.0001; T_48_ vs T_0_, p-value <0.0001). ΔCq = (Cq_biomarker_ – Cq_meanref_) with Cq_meanref_ = mean of the Cq values of the 3 reference genes.

**Figure 2:**
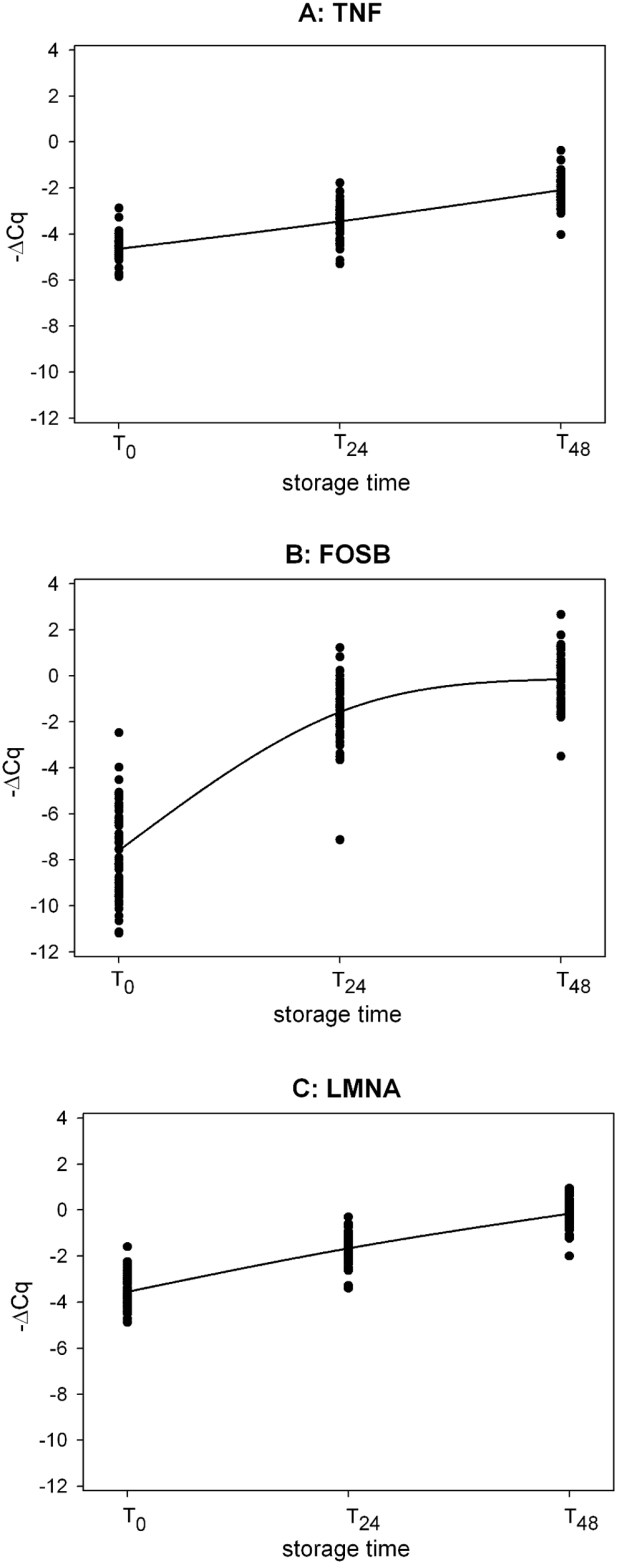
Time-course profile of EDTA up-regulation biomarkers in the validation study. 2A: TFN_S (mixed model contrasts: T_24_ vs T_0_, p-value <0.0001; T_48_ vs T_0_, p-value <0.0001); 2B: FOSB_S (mixed model contrasts: T_24_ vs T_0_, p-value <0.0001; T_48_ vs T_0_, p-value <0.0001); 2C: LMNA_S (mixed model contrasts: T_24_ vs T_0_, p-value <0.0001; T_24s_ vs T_0_, p-value <0.0001). ΔCq = (Cq_biomarker_ – Cq_meanref_).

**Figure 3:**
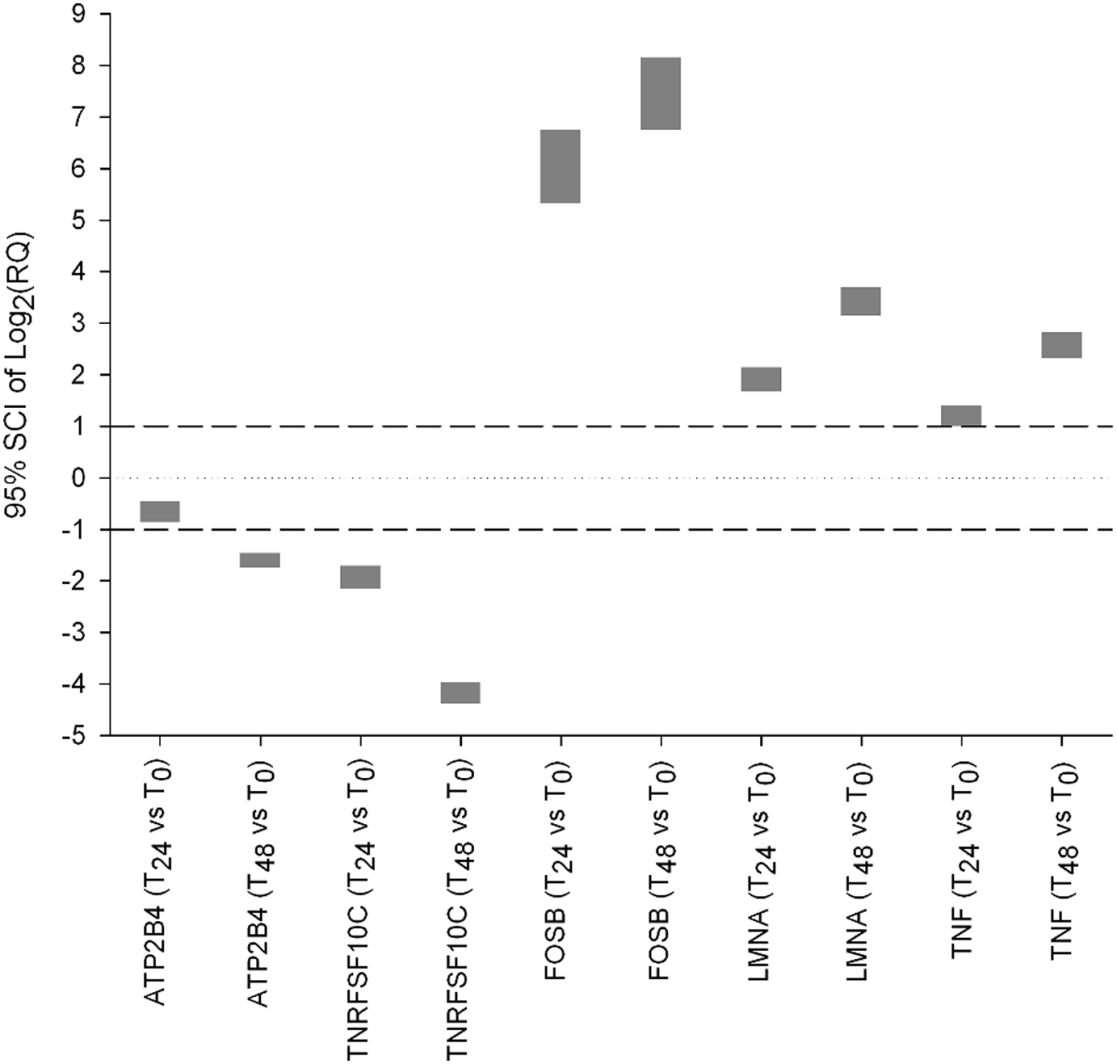
95% Simultaneous Confidence Intervals (SCIs) of the Log_2_ Relative Quantity (RQ) for the EDTA biomarkers. For each time point T_x_ (x≠0) the corresponding RQ was computed as 2^−[ΔΔCq]^ where ΔΔCq = ΔCq marker_Tx_−ΔCqmarker_T0_.

For EDTA down-regulation biomarkers, ATP2B4 and TNRFSF10C, and up-regulation biomarkers, LMNA, TNF, and FOSB, a significant change in expression was observed between T_0_ and T_24_ and T_48_ (Figure 1, 2). Furthermore, for each EDTA biomarker, we evaluated the relevance of the expression changes between T_0_ and the other two time points by computing the 95% simultaneous confidence interval (SCI) of the Log_2_ of the relative quantity (RQ). For biomarkers LMNA, FOSB and TNRFSF10C, the 95% SCIs showed significant changes from T_0_ in gene expression for both time points tested. For the other biomarkers, ATP2B4 and TNF, these changes were less pronounced, especially at T_24_ (Figure 3).

**Figure 4:**
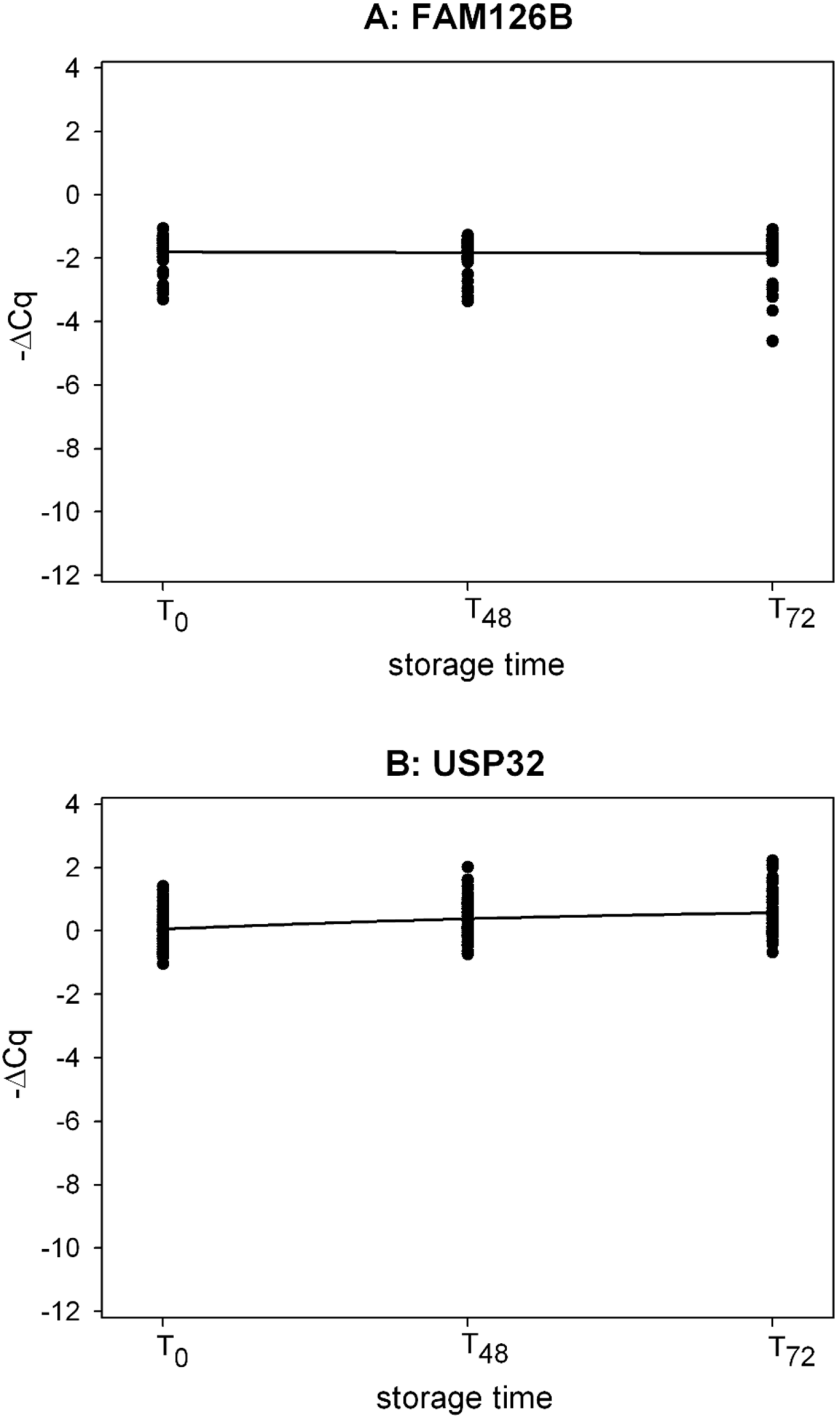
Time-course profile of the PAXgene biomarkers in the validation study. 4A: FAM126B (mixed model contrasts: T_48_ vs T_0_, p-value = 0.5251; T_72_ vs T_0_, p-value = 0.2734) and 4B: USP32 (mixed model contrasts: T_48_ vs T_0_, p-value <0.0001;T_72_ vs T_0_, p-value<0.0001). ΔCq = (Cq_short assay_−Cq_medium assay_).

For PAXgene biomarkers (FAM126B and USP32), observable changes were only evident for USP32 in the S/M ratio between T_0_ and T_48_/T_72_. For FAM126B, the S/M ratio did not change significantly in PAXgene tubes even when blood was stored at 30°C (Figure 4).

In summary, all EDTA quality biomarker assays were validated. Among these, FOSB was found to be the most relevant (highest fold-change between T_0_ and the other two time points) followed by TNRFSF10C and LMNA. In addition the multivariate analysis performed by jointly considering these three quality biomarkers (data not shown) revealed that the power of detection of FOSB was not enhanced by adding the other two biomarkers. For the PAXgene biomarkers, only USP32 showed significant changes in expression between T_0_ and later time points and then only at elevated temperatures. All information on validated biomarkers is summarized in Table S1.

## Discussion

It is increasingly recognized that pre-analytical factors, if not properly recognized and controlled [29]–[31], can have an effect on sample quality and, consequently, on the quality of molecular analysis. This is particularly true for sensitive analytical molecular methods like qPCR. With the current increasing focus on molecular and companion diagnostics, the development and implementation of adequate quality control tools for all steps of the process is critical for clinical success.

Here, we focus on the *ex* *vivo* changes in gene expression in blood specimens [3], [4]. The main option for minimizing these pre-analytical effects include the immediate stabilization of blood RNA using special collection tubes such as PAXgene tubes [32] or Tempus Blood RNA tubes (Applied Biosystems, Foster City, CA) [33], [34]. These tubes provide a better alternative to traditional blood collection tubes containing K_2_EDTA or heparin which render the transcripts vulnerable to degradation and dysregulation. To date, no reliable biomarkers of RNA quality in collected blood specimens have been described. This makes the evaluation of methods for preserving RNA quality in clinical specimens a challenge.

We have developed and validated quality biomarkers that can detect up- (FOSB, LMNA) and down-regulation/degradation (TNRFSF10C) of mRNA in blood collected in EDTA tubes and mRNA degradation at high temperatures (30°C) of in PAXgene tubes (USP32).

EDTA tubes, while widely used, do not contain any stabilizer of gene expression [35]. Indeed, it has been shown that EDTA does not prevent changes of gene expression even during short-term storage [32], [36]. Dysregulated transcripts in blood in EDTA tubes have been identified previously, for example IL8, TNF, IFNG or ICAM [32] and have been used to demonstrate the need for stabilization of blood samples for transcript analyses [32], but none of these transcripts has been validated as biomarker for the assessment of mRNA quality in blood. This study is the first one, in which stability of transcripts in blood has been systematically approached and relevant biomarkers identified and validated using enough individual samples to attain sufficient statistical power.

In the microarray analysis, we identified 2.4 times more candidates for up- or down-regulated transcripts for blood incubated in EDTA tubes than that in PAXgene tubes at RT. As expected, there was a higher rate of degradation and dysregulation observed in EDTA blood samples between T_0_ and T_48_. In addition, other significant changes in gene expression were detectable earlier in EDTA tubes and, in the case of FOSB, after only 2 h at RT (see Figure S2, panel C). The other two validated biomarkers for EDTA tubes detected dysregulation of gene expression after 24 h at RT. TNRFSF10C detected down-regulation, and LMNA detected up-regulation.

PAXgene tubes, which are designed to stabilize blood RNA, contain a specific additive that lyses the blood cells and stabilizes the RNA *in* *situ* for up to 3 d at 18–25°C, 5 d at 2–8°C, and at least 6 years at −20–−80°C. While it was not possible to find a biomarker that showed significant degradation effects under these conditions, and there was no evidence of dysregulation in PAXgene tubes, RNA could be chemically degraded with time because RNA *per se* is a labile molecule [37]. To test this hypothesis, we identified and validated one biomarker for RNA degradation in PAXgene tubes, USP32. Interestingly, using this biomarker, RNA degradation was only evident after blood was stored in PAXgene tubes at 30°C for several days. This suggested that some level of RNA degradation can occur in blood stored in PAXgene tubes but only under off-label conditions. Therefore, for the final validation of the biomarker, an increased storage temperature of 30°C was used to simulate an extreme situation for which even the PAXgene chemistry cannot guarantee the stabilization of all transcripts. Such markers were meant also as quality markers to monitor stabilized blood samples, when, for instance, the transport conditions are not controlled and there is a risk for high temperature exposure of the samples.

We are aware of some limitations of the preset study. Suggested biomarkers have been validated in the healthy population. In research and clinical settings, samples are mostly collected from patients. In these subjects, disease mechanisms could bring potential variation on the level of the biomarkers. We have tried to limit the potential disease regulated effect by selecting biomarker candidates that are known not to be involved in any disease pathways. However, the effect of other confounding factors e.g. medication or hypoxia has not been tested. Therefore, the biomarkers should be validated for the given disease or the specific condition before use.

In addition, we validated specific biomarkers separately for EDTA and PAXgene tubes, and since each pre-analytical method may be biased in certain ways, these biomarkers are not suitable for use with other blood collection tubes, for example the Tempus tube (Life Technologies) or EDTA biomarkers cannot be used as quality biomarkers for PAXgene tubes.

How to use our biomarkers for validation of pre-analytical experimental workflow? Sample quality can be measured by comparing T_0_ reference sample with tested sample. Eventually, other time points could be included. This could represent the first step to evaluate if different pre-analytical conditions have a significant impact on the quality of the tested transcripts.

Importantly, the practical use of our validated biomarkers was demonstrated in the 2^nd^ ring trial of the SPIDIA-RNA Program in which the effects of pre-analytical procedures on RNA quality were evaluated in 109 European clinical and research laboratories (manuscript submitted for publication, “SPIDIA-RNA: Second External Quality Assessment for the pre-analytical phase of blood samples used for RNA based analyses”). The performance of the participating laboratories was tested by their RNA preparation from stabilized and unstabilized blood specimens. The extracted RNAs were analysed and compared to T_0_ in SPIDIA reference laboratory by using traditional procedures as evaluation of RNA purity, integrity, testing the presence of inhibitors and qPCR evaluation of differentially expressed transcripts FOS, IL1β, IL8 and GAPDH. In addition, two our validated biomarkers, FOSB and TNFRS, which indicated ex vivo gene expression changes in stored blood, were used to determine the extent of gene transcription instability in stabilized and unstabilized blood specimens.

The results from this comprehensive evaluation demonstrated that these biomarkers can be used as quality control tools for the pre-analytical workflow of blood samples used for RNA-based analyses.

## Supporting Information

Figure S1 Agarose gel electrophoresis of PCR products for the 63 designed qPCR assays.
(PDF)

Figure S2 Pre-validation of up-regulated EDTA biomarkers.
(PDF)

Figure S3 Pre-validation of down-regulated EDTA biomarkers.
(PDF)

Figure S4 Pre-validation of the PAXgene degradation biomarker FAM126B.
(PDF)

Figure S5 Pre-validation of the PAXgene degradation biomarker USP32.
(PDF)

Figure S6 Overall standard deviations for all assays.
(PDF)

Table S1 qPCR assay design information for all biomarkers.
(XLSX)

Table S2 Biomarker precision analysis.
(PDF)

File S1 Microarray data set.
(XLSX)

File S2 Pre-validation and precision study.
(DOCX)
