# Supplementary material for: Biomarkers for Monitoring Pre-Analytical Quality Variation of mRNA in Blood Samples
Source: PLoS One. 2014 Nov 4;9(11):e111644. doi: 10.1371/journal.pone.0111644 (PMC4219744; doi:10.1371/journal.pone.0111644)
Supplement: Figure S2 — Pre-validation of up-regulated EDTA biomarkers. (PDF) [file pone.0111644.s002.pdf]

Figure S2. Pre-validation of up-regulated EDTA biomarkers.

The figure reports the distributions over time of the –DeltaCq of up-regulated markers in six EDTA samples. Where DeltaCq =($Cq_{biomarker}$ – Cq_{mref}) and Cq_{mref} is the mean of the Cq values of the 3 reference genes. In the tables are reported the p-value of the contrast implemented in the ANOVA mixed model.

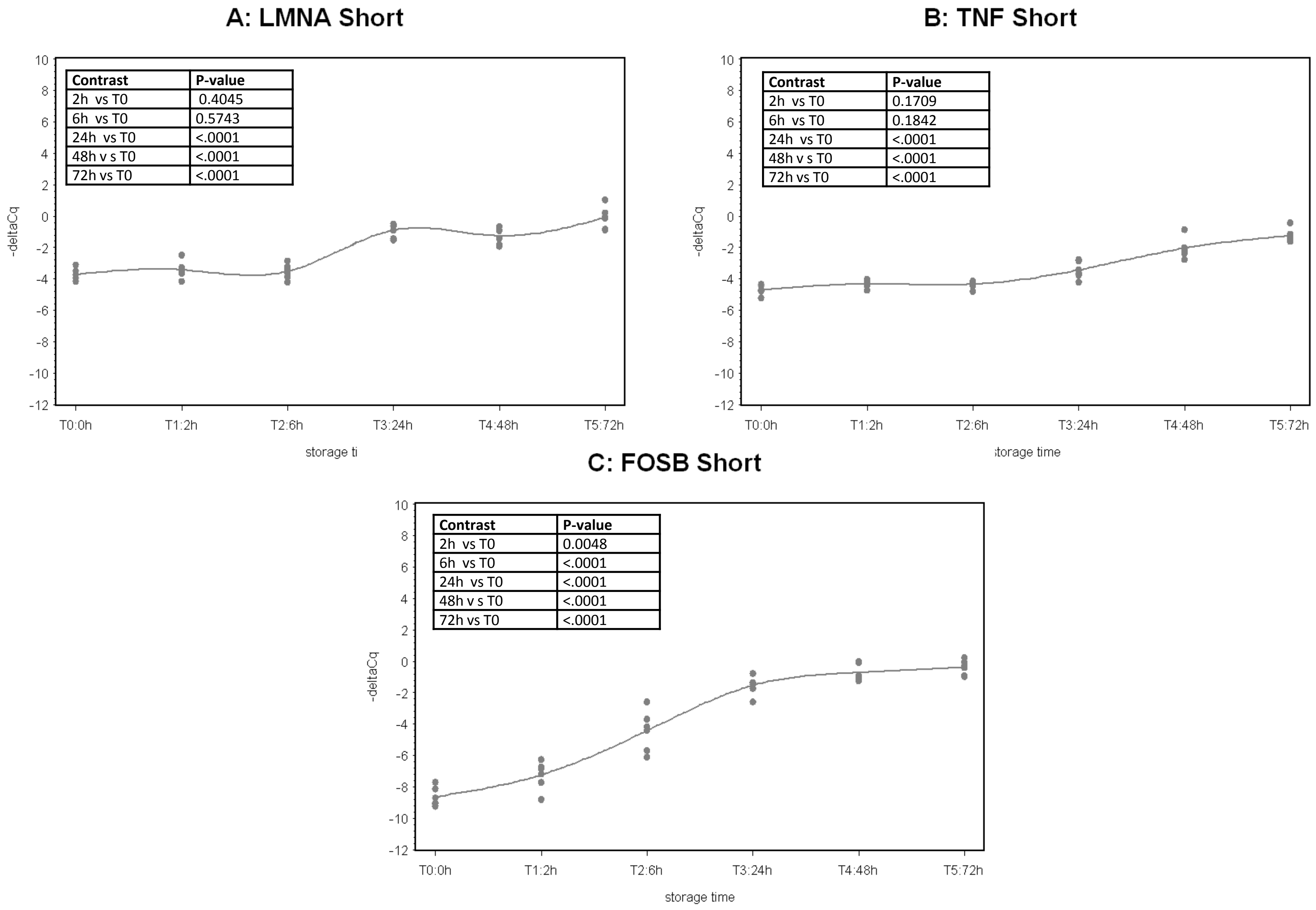

A: LMNA Short

Contrast	P-value
2h vs T0	0.4045
6h vs T0	0.5743
24h vs T0	<.0001
48h v s T0	<.0001
72h vs T0	<.0001

B: TNF Short

Contrast	P-value
2h vs T0	0.1709
6h vs T0	0.1842
24h vs T0	<.0001
48h v s T0	<.0001
72h vs T0	<.0001

C: FOSB Short

Contrast	P-value
2h vs T0	0.0048
6h vs T0	<.0001
24h vs T0	<.0001
48h v s T0	<.0001
72h vs T0	<.0001
