# Supplementary material for: Biomarkers for Monitoring Pre-Analytical Quality Variation of mRNA in Blood Samples
Source: PLoS One. 2014 Nov 4;9(11):e111644. doi: 10.1371/journal.pone.0111644 (PMC4219744; doi:10.1371/journal.pone.0111644)

Figure S3. Pre-validation of down-regulated EDTA biomarkers.

The figure reports the distributions over time of the –*Delta*Cq of down-regulated markers in six EDTA samples. Where DeltaCq =($Cq_{biomarker}$ – Cq_{mref}) and Cq_{mref} is the mean of the Cq values of the 3 reference genes. In the tables are reported the p-value of the contrast implemented in the ANOVA mixed model.

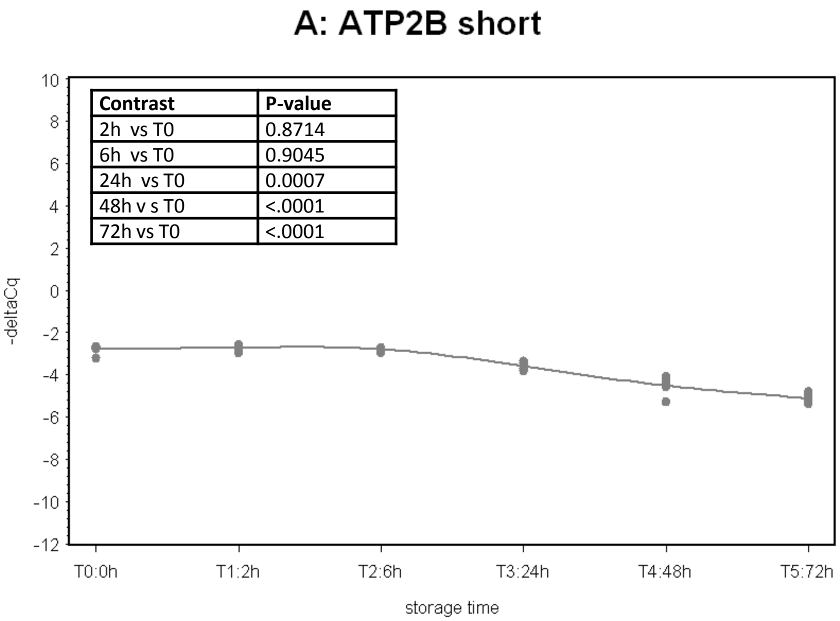

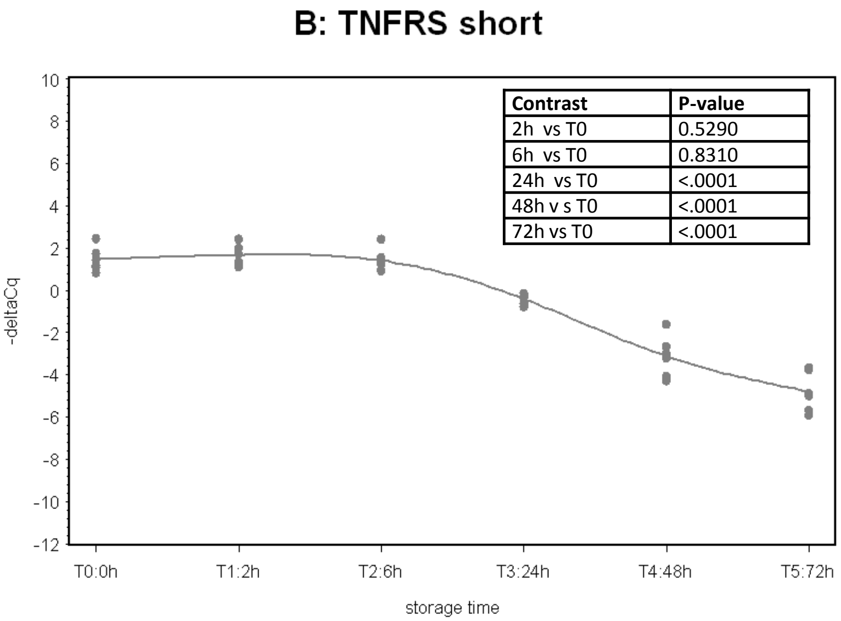

Supplement: Figure S3 — Pre-validation of down-regulated EDTA biomarkers. (PDF) [file pone.0111644.s003.pdf]
