# Supplementary material for: Biomarkers for Monitoring Pre-Analytical Quality Variation of mRNA in Blood Samples
Source: PLoS One. 2014 Nov 4;9(11):e111644. doi: 10.1371/journal.pone.0111644 (PMC4219744; doi:10.1371/journal.pone.0111644)
Supplement: Figure S4 — Pre-validation of the PAXgene degradation biomarker FAM126B. (PDF) [file pone.0111644.s004.pdf]

Figure S4. Pre-validation of the PAX degradation biomarkers FAM126B.

The figure reports the distributions over time of the –*Delta*Cq 3’5’ and –*Delta*Cq S/M of FAM126B biomarkers in the 8 PAX samples stored at RT and in the 5 PAX samples stored at 35°C. Where DeltaCq 3’5’ = (Cq 3’– Cq 5’) and DeltaCq S/M = Cq Short – Cq Medium) In the tables are reported the p-value of the contrast implemented in the ANOVA mixed model.

A: FAM 3'/5' at RT

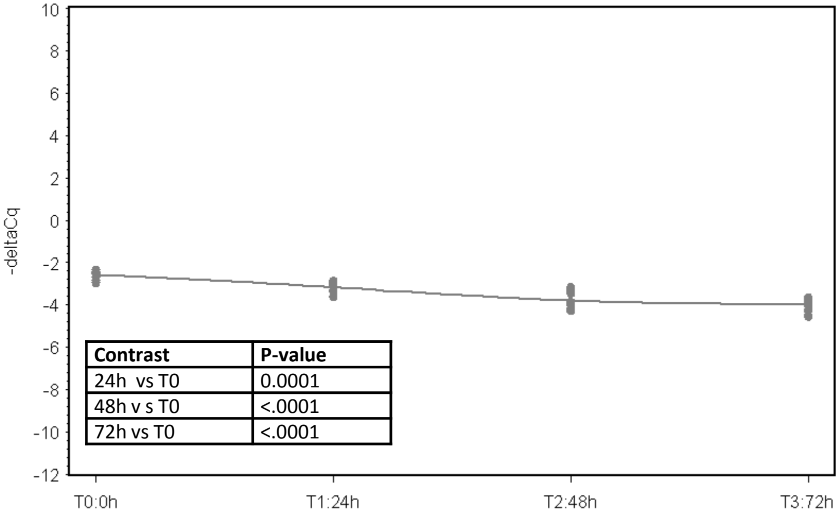

Contrast	P-value
24h vs T0	0.0001
48h v s T0	<.0001
72h vs T0	<.0001

B: FAM 3'/5' at 35°C

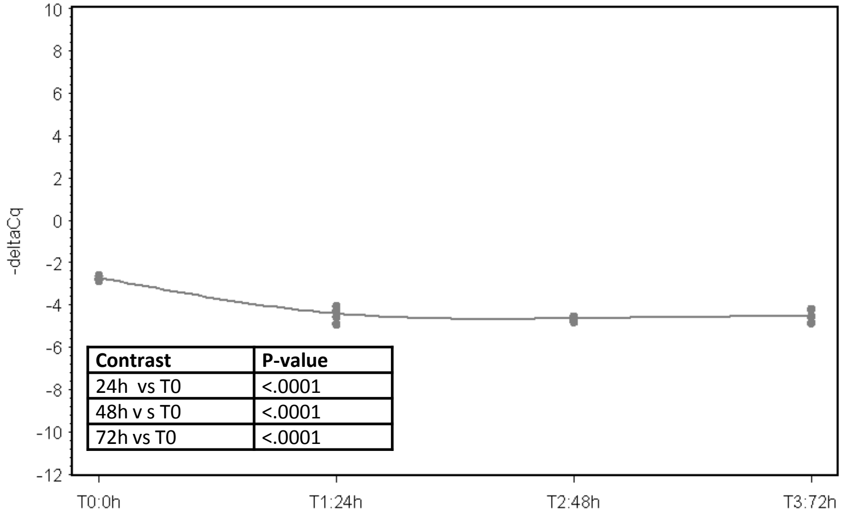

Contrast	P-value
24h vs T0	<.0001
48h v s T0	<.0001
72h vs T0	<.0001

C: FAM S/M at RT

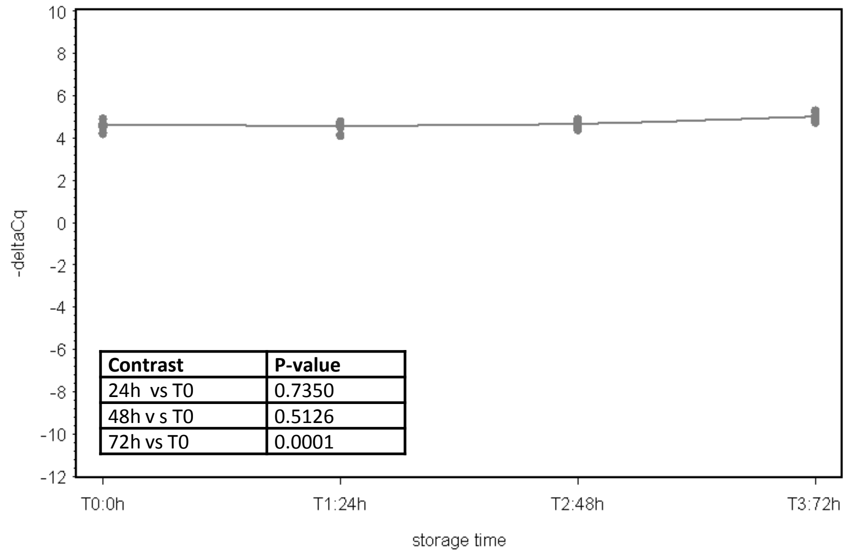

Contrast	P-value
24h vs T0	0.7350
48h v s T0	0.5126
72h vs T0	0.0001

D: FAM S/M at 35°C

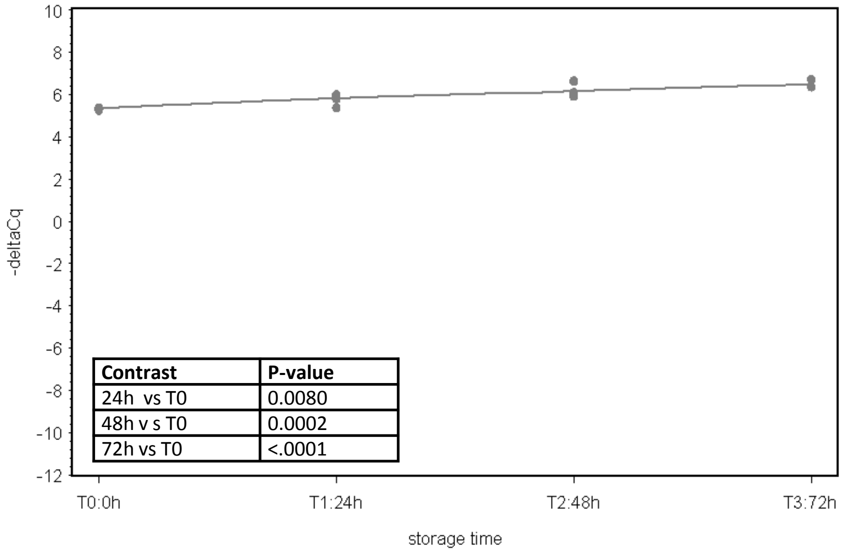

Contrast	P-value
24h vs T0	0.0080
48h v s T0	0.0002
72h vs T0	<.0001
