# Supplementary material for: Biomarkers for Monitoring Pre-Analytical Quality Variation of mRNA in Blood Samples
Source: PLoS One. 2014 Nov 4;9(11):e111644. doi: 10.1371/journal.pone.0111644 (PMC4219744; doi:10.1371/journal.pone.0111644)

**Figure S5. Pre-validation of the PAX degradation biomarkers USP32.**

The figure reports the distributions over time of the –DeltaCq 3’5’ and –*Delta*Cq S/M of USP32 biomarkers in the 8 PAX samples stored at RT and in the 5 PAX samples stored at 35°C. Where DeltaCq 3’5’ = (Cq 3’– Cq 5’) and DeltaCq S/M = Cq Short – Cq Medium) In the tables are reported the p-value of the contrast implemented in the ANOVA mixed model.

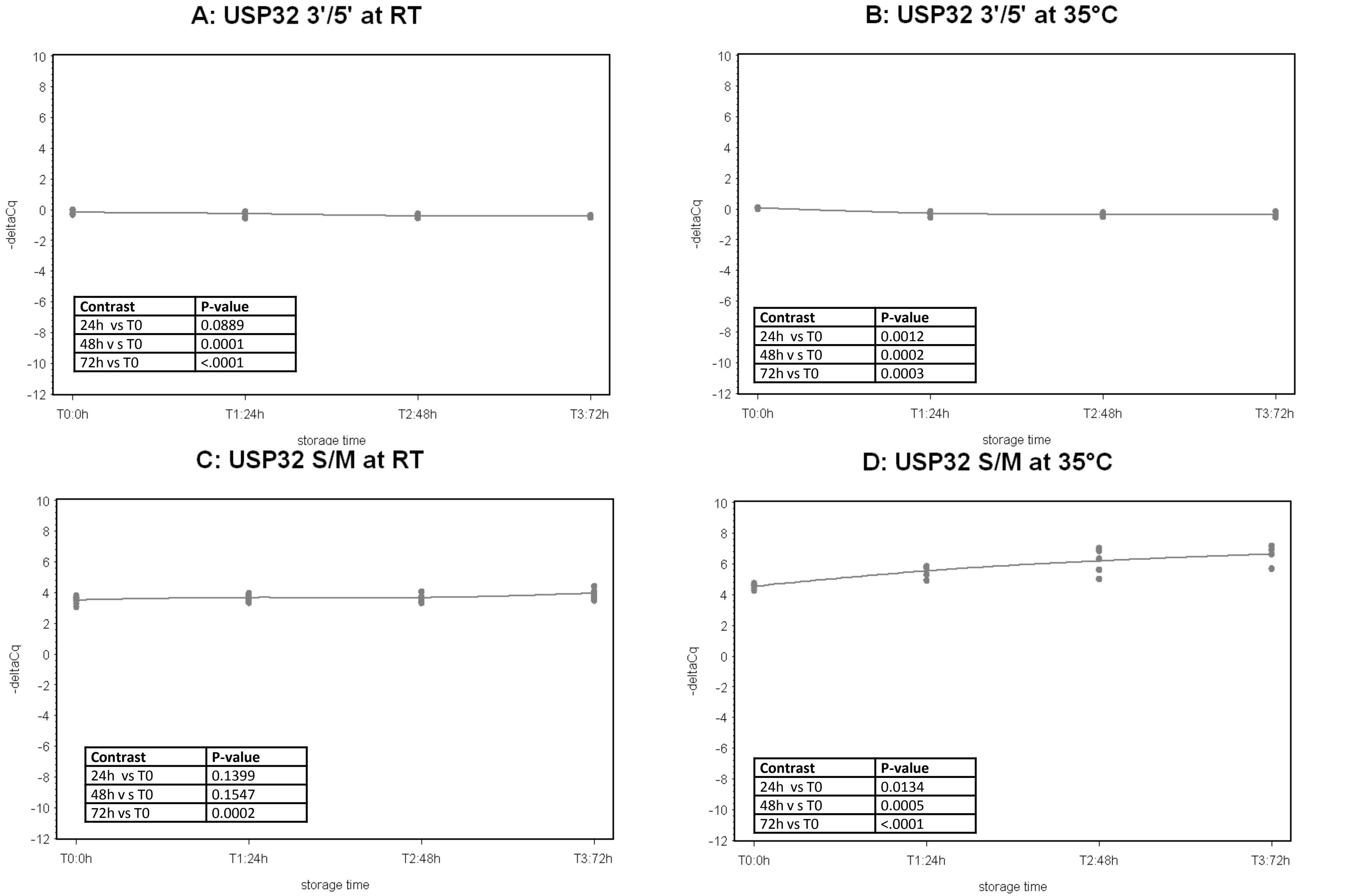

Supplement: Figure S5 — Pre-validation of the PAXgene degradation biomarker USP32. (PDF) [file pone.0111644.s005.pdf]
