# Supplementary material for: Biomarkers for Monitoring Pre-Analytical Quality Variation of mRNA in Blood Samples
Source: PLoS One. 2014 Nov 4;9(11):e111644. doi: 10.1371/journal.pone.0111644 (PMC4219744; doi:10.1371/journal.pone.0111644)

Figure S6. *Overall standard deviations for all assays. SD of combined day and lab effect (Y-axis) was calculated for all assays, including 5' or 3' assay (5p or 3p), short (S), medium (M), and long (L) assays (X-axis).*

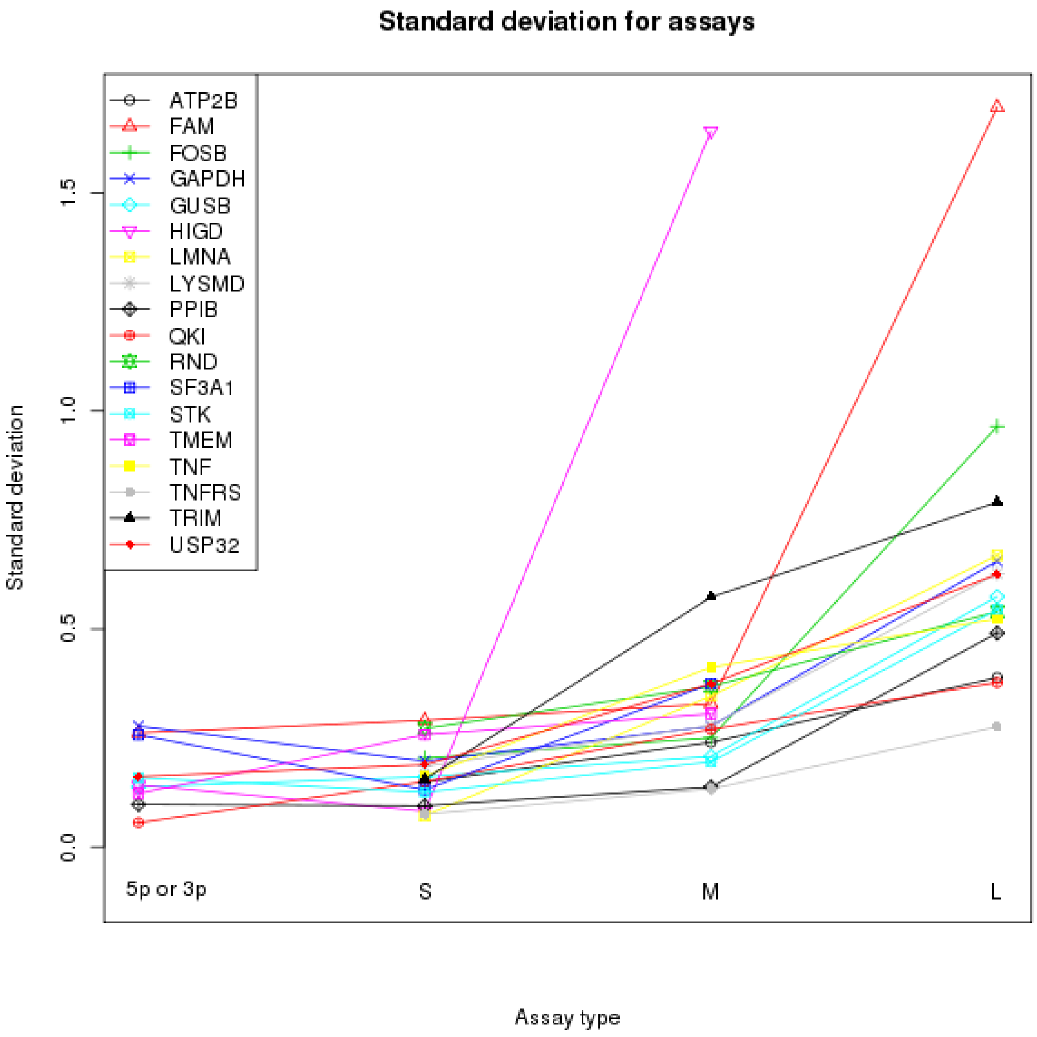

Supplement: Figure S6 — Overall standard deviations for all assays. (PDF) [file pone.0111644.s006.pdf]
