# Supplementary material for: Biomarkers for Monitoring Pre-Analytical Quality Variation of mRNA in Blood Samples
Source: PLoS One. 2014 Nov 4;9(11):e111644. doi: 10.1371/journal.pone.0111644 (PMC4219744; doi:10.1371/journal.pone.0111644)
Supplement: Table S2 — Biomarker precision analysis. (PDF) [file pone.0111644.s008.pdf]

Table S2. Biomarker precision analysis

Biomarker	Assay	Ratio ($\Delta\Delta$Cq)	Precision CI (95%)	Accepted precision
FAM126B	S/M	0.85	0.34	yes
	5’/3’	2.28	0.37	yes
USP32	S/M	0.74	0.29	yes
	5’/3’	0.73	0.38	yes
GAPDH	S/M	0.14	0.30	yes
	5’/3’	0.21	0.39	yes
GUSB	S/M	0.45	0.38	no
	5’/3’	0.59	0.35	no
PPIB	S/M	0.03	0.29	yes
	5’/3’	0.01	0.14	yes
LMNA	S	2.38	0.07	yes
	M	2.48	0.30	yes
TNF	S	1.40	0.11	yes
	M	1.39	0.27	yes
FOSB	S	7.67	0.15	yes
	M	7.48	0.17	yes
ATP2B	S	-1.16	0.10	yes
	M	-1.35	0.17	yes
TNFRS	S	2.37	0.06	yes
	M	2.51	0.10	yes
