# Supplementary material for: Biomarkers for Monitoring Pre-Analytical Quality Variation of mRNA in Blood Samples
Source: PLoS One. 2014 Nov 4;9(11):e111644. doi: 10.1371/journal.pone.0111644 (PMC4219744; doi:10.1371/journal.pone.0111644)
Supplement: File S2 — Pre-validation and precision study. (DOCX) [file pone.0111644.s010.docx]

**Supplementary materials for “Biomarkers for monitoring pre-analytical quality variation of RNA in blood samples”**

**Pre-validation and precision study.**

**Materials and Methods**

Sample material and RNA extraction methods for pre-validation and precision study are described in the paper.

The procedure for RNA extraction from EDTA blood with acidic organic phenol extraction and silica membrane clean-up is described here. Two ml of EDTA blood was mixed with 6 ml TRIzol®LS reagent (Life Technologies) and 2 ml chloroform in a screw cap tube and centrifuged for 15 min at 12.000 xg for phase separation. The aqueous phase was transferred to a new tube and 0,5 vol. of ethanol (96% (v/v)) was added. The sample was applied to a RNeasy® Mini spin column in aliquots of 700 µl via centrifugation at 8.000-20.000 x g for 1 min. The RNA was purified, including the on column DNase digestion step, according to the RNeasy Mini Kit handbook manufacturer’s instructions (QIAGEN, Hilden). The RNA was eluted in 80 µl RNase free water.

**Statistical analysis**

(i) Pre-validation study

For the analysis of the pre-validation data, a mixed model ANOVA (1) was implemented by evaluating dependent variable -ΔCq as a function of ‘time’ (fixed factor)‘donor’ (random factor). For the up- and down-regulated EDTA biomarkers, only the Cq values of short

(amplicon size: 80 -160 bp) assays were utilized for the computation of the corresponding –ΔCq at each time point as follows: ΔCq marker = Cq marker – Cq meanref, where Cq meanref is the mean of the Cq values derived from the short assay of three reference genes identified in the microarray analysis: GAPDH, GUSB, and PPIB.

For the PAXgene degradation biomarkers, the Cq values of the 3’ and 5’ assays, as well as those of the short and medium (amplicon size: 200- 370 bp) assay were utilized for the calculation of the corresponding ΔCq at each time point as follows:

ΔCq marker S/M = Cq short assay – Cq medium assay

ΔCq marker 3´/5´ = Cq 3’ assay – Cq 5’ assay

The analysis of the pre-validation study was carried out using SAS software v. 9.2 (SAS Institute Inc. Cary, NC)

(ii) Precision analysis

For the precision analysis, the overall precision for each assay was calculated as repeatability (standard deviation, SD) of the Cq values across the three days (Day), two laboratories (Lab) and the two incubation time points (Time). A mixed model ANOVA was used to determine the size of the effects (Day, Lab and Time) in the study. Day and Lab were modeled as random effects, and Time was modeled as fixed effect.

For up- and down-regulated biomarkers, the Cq values of short assays and medium assays were considered as dependent variables in the model. For the degradation and reference biomarkers, the Cq values of the 3’ and 5’ assays, as well as those of the short and medium assays were utilized for the calculation of the corresponding ΔCq as indicated above. The latter was considered as dependent variables in the model.

The lme4 package for R (2) was used to perform the calculation. For up- and down-regulated biomarkers, the fixed effect of time and the reported Standard Error (SE) of this estimate were used to validate the biomarker. If the size of the time effect (ΔCq=Cq_T48_-Cq_T0_) was larger than z*SE, the marker was considered as validated for precision. Where z correspond to a prefixed centile of the Standard Normal Distribution

For validating the degradation and reference biomarkers, the same mixed ANOVA model was adopted. In this case, because the dependent variable is the ΔCq of the assays, the resulting size of the time effect can be defined as: ΔΔCq S/M =ΔCq_T48_ S/M - ΔCq_T0_ S/M; ΔΔCq 3’/5’ =ΔCq_T48_3’/5’ - ΔCq_T0_ 3’/5’). By following the same approach described above, the size of the time effect and its standard error were estimated, and if the effect was larger than z*SE, the degradation marker was reported as validated for precision. By the same token, a reference marker was validated if the effect was less than z*SE.

**Results**

**Pre-validation study of RNA quality biomarkers**

In order to demonstrate whether the identified biomarker candidates could detect RNA degradation and dysregulation, qPCR assays were designed and pre-validated using blood samples from 5-8 healthy volunteers (Table 1 in the paper). A total of 63 assays were designed and optimized for these RNA biomarker candidates, and reference genes (a total of 18 transcripts, Table 2 in the paper), including 3’/5’ assays and long-medium-short assays for potential degradation markers and reference genes, and only long-medium-short assays for up-/down-regulation markers. Assay specificities were evaluated by the gel electrophoresis of amplified products (Figure S1) and assay linear range and sensitivities were determined by a serial dilution of the target amplicon. All assays performed well in the range 2 million to 200 copies per PCR with the exception of assay GUSB long that performed well in the range 2 million to 2000 copies per PCR. Only short and medium assays were considered for data analysis, since the long (amplicon size: 400 – 550 bp) assays were shown to have poor performance in the pre-validation study.

The time points for incubation of EDTA tubes at room temperature (RT) were 0, 2, 6, 24, 48 and 72 hours. Among four tested EDTA up-regulation markers, LMNA, TNF and FOSB demonstrated a significant (p<0.05) up-regulation after 24-72h hours (Figure S2) with respect to T_0_. FOSB, an immediate early response gene, was significantly up-regulated after 2 h. The EDTA down-regulation markers, TNFRS and ATP2B, were significantly altered after 24-72 hours (Figure S3) with respect to T_0_. Since it is difficult to distinguish between down-regulation and transcript degradation, the down-regulation biomarkers can be considered as overall markers for decreased transcript levels in EDTA samples.

We have observed that RNA degradation is a very slow process in blood stored at RT (18 – 25^o^C) in PAXgene tubes . In contrast to EDTA tubes, PAXgene tubes contain a special additive that stabilizes the *in vivo* gene transcription profile by reducing *in vitro* RNA degradation and minimizing gene induction (3). Thus, to be able to demonstrate significant degradation in blood collected in PAXgene tubes, samples were stored at both RT and 35°C for 24, 48 and 72 hours and compared to samples kept at RT for 2 h (T_0_). The pre-validation study demonstrated that the FAM126B 3’/5’ biomarker assay showed the highest ratio and a greatest difference between the time points with respect to T_0_ both at 35°C and at RT (Figure S4). For the FAM126B S/M biomarker assay, the difference between later time points and T_0_ were significant after 24h-72h only at 35°C, whereas at RT, the difference was significant after 72 hours. USP32 S/M and 3’/5’ biomarker assays showed lower ratio changes, even though the changes were significant (Figure S5) after 24h-72h at 35°C and after 48 (for 3’/5’) or 72h (for S/M) at RT. Of note was the fact that it was much more challenging to develop quality biomarkers for blood stored in PAXgene tubes than for blood stored in EDTA tubes. This is most likely due to fewer transcript expression changes over time observed in blood collected in PAXgene tubes than in blood collected in EDTA tubes.

**Precision analysis**

The overall precision is reported for each assay expressed as the standard deviation of the Cq values across the three days, two laboratories and the two incubation times (figure S6). Short assays showed better precision than the medium and long assays. Most of the long assays exhibited high SDs, which explains why most S/L ratios could not be calculated in the pre-validation study.

The precision validations of the time effect of the biomarkers as well as of the reference biomarkers were performed by using z=1.96 which corresponds to the 97.5th centile of the Standard Normal Distribution. The results are displayed in the Table S1. The time effects of both FAM126B and USP32 ΔCq values are more than 1.96 times higher than the standard error indicating acceptable precision. For all EDTA up- and down-regulated biomarkers, the time effect of Cq values are also more than 1.96 times higher than the standard deviation which indicates acceptable precision.

Since the validity of the EDTA up- and down-regulated biomarkers depended on the reliability of the reference genes used for normalization, we also explored the level of degradation of the reference genes GAPDH, GUSB and PPIB in EDTA blood samples. For a reference biomarker to be considered intact, the time effect of ΔCq values should be less than 1.96 times the standard error. Both GAPDH and PPIB were stable over 48 hours with acceptable precision, whereas GUSB showed larger ΔCq changes than 1.96 times the standard error even though the ΔCq changes were small (0.45 for S/M and 0.59 for 3’/5’). Therefore, GUSB was not excluded from the reference biomarker panel.

**References**

1. McCulloch C, Searle S. Generalized, linear and mixed models. New York: John Wiley & Sons, Inc., 2001.
2. Bates D, Maechler M, Bolker B, Walker S. Lme4: Linear mixed-effects models using eigen and s4. http://CRAN.R-project.org/package=lme4 (Accessed 2013).
3. PreAnalytiX. Paxgene blood rna tube. http://www.preanalytix.com/product-catalog/blood/rna/products/paxgene-blood-rna-tube/ (Accessed 29/08/2013 2013).
