# Supplementary material for: Biomarkers for Monitoring Pre-Analytical Quality Variation of mRNA in Blood Samples
Source: PLoS One. 2014 Nov 4;9(11):e111644. doi: 10.1371/journal.pone.0111644 (PMC4219744; doi:10.1371/journal.pone.0111644)

Figure S1. Agarose gel electrophoresis (Lonza FlashGel[TM] 2.2% DNA Cassette) of PCR products for the 63 designed qPCR assays. A DNA size ladder (Lonza FlashGel[TM] 50-1500bp DNA marker) is shown in lanes 1, 14, 27 and 40.

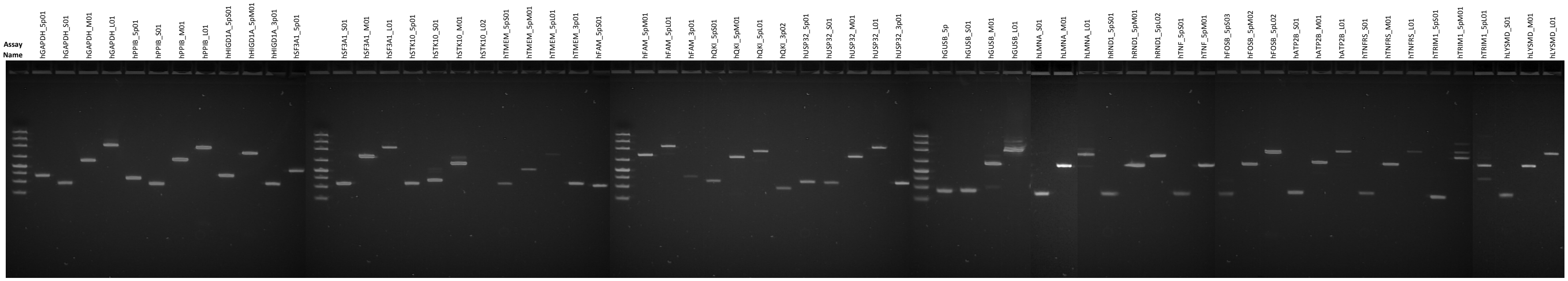

Supplement: Figure S1 — Agarose gel electrophoresis of PCR products for the 63 designed qPCR assays. (PDF) [file pone.0111644.s001.pdf]
